# Synergy between Experimental and Theoretical Results of Some Reactions of Annelated 1,3-Azaphospholes

**DOI:** 10.3390/molecules23061283

**Published:** 2018-05-27

**Authors:** Raj K. Bansal, Raakhi Gupta, Manjinder Kour

**Affiliations:** Department of Chemistry, The IIS University, Jaipur 302020, India; raakhi.gupta@iisuniv.ac.in (R.G.); manjinderkour1992@gmail.com (M.K.)

**Keywords:** synergy, 1,3-azaphospholes, Diels-Alder reaction, electrophilic substitution, DFT calculations

## Abstract

Computational calculations have been used successfully to explain the reactivity of the >C=P- functionality in pyrido-annelated 1,3-azaphospholes. Theoretical investigation at the Density Functional Theory (DFT) level shows that the lone pair of the bridgehead nitrogen atoms is involved in extended conjugation, due to which electron density increases considerably in the five-membered azaphosphole ring. The electron density in the azaphosphole is further enhanced by the presence of an ester group at the 3-position making the >C=P- functionality electron-rich. Thus, 1,3-azaphospholo[5,1-*a*]pyridine, i.e., 2-phosphaindolizine having ester group at the 3-position only does not undergo Diels-Alder (DA) reaction with an electron rich diene, such as 2,3-dimethyl-1,3-butadiene (DMB). However, an ester group at 1-position acts as electron-sink, due to which transfer of the electron density to the >C=P- moiety is checked and DA reaction occurs across the >C=P- functionality. The coordination of the Lewis acid to the carbonyl group at the 3-position raises the activation barrier, while it is lowered remarkably when it is coordinated to the P atom. Furthermore, the attack of 1,3-butadiene on the *Si* face of the P-coordinated (*o*-menthoxy)aluminum dichloride complex is a lower activation energy path. Fukui functions could be used to explain relative reactivities of indolizine and 2-phosphaindolizine towards electrophilic substitution reactions.

## 1. Introduction

Almost a century ago, Paul Dirac [1] could foresee the possibility of applying quantum mechanical concepts to solving the experimental problems in chemistry when he remarked, “The underlying physical laws necessary for the mathematical theory of a large part of physics and the whole of chemistry are thus completely known, and the difficulty is only that the exact application of these laws leads to equations much too complicated to be soluble. It therefore becomes desirable that approximate practical methods of applying quantum mechanics should be developed, which can lead to an explanation of the main features of complex atomic systems without too much computation”. Dirac’s dream inched towards fruition when Kohn, Hohenberg, and Sham [2,3] developed the density functional theory (DFT). It was followed by dramatic progress when many DFT codes were made available commercially, which enabled even less experienced persons with modest computational facilities to do calculations with much accuracy and look into the electronic structures and many other properties of the substances of interest. During the last two decades, the availability of a variety of new and more accurate DFT functionals incorporating specific features has brought DFT computations to the stage where they are used advantageously as a tool not only to explain the experimental results but also to plan and modify experimental conditions for achieving the desired objectives [4,5,6,7,8,9]. In recent years, there has been a perceptible increase in the number of research papers in chemistry reporting both experimental and theoretical results together. Fristrup and co-workers in a recent article emphasized the synergy between experimental and theoretical methods in the exploration of homogeneous transitional metal catalysis [10]. In another interesting review article titled, “Towards the computational design of solid catalysts”, Norskov et al. [11] commented, “*Theoretical methods can be used to describe surface chemical reactions in detail and to understand variations in catalytic activity from one catalyst to another*.” In spite of many challenges in the field, they added “Computational approaches for the discovery and development of catalysts hold great promise for the future.” Similar views have been expressed in other reports [12,13,14].

In this perspective, we shall present mainly our own results highlighting the application of DFT calculations to plan and accomplish successfully some reactions of annelated 1,3-azaphospholes. It will also be illustrated how computational calculations helped to resolve an experimental log jam and paved the way for the use of catalysts, leading to a successful reaction and asymmetric synthesis.

## 2. Synthesis of Annelated 1,3-Azaphospholes

Before coming to the reactions, it will be appropriate to describe briefly two general methods of synthesis of annelated 1,3-azaphospholes that we developed.

### 2.1. [4+1] Cyclocondensation Method

**Scheme 1:**
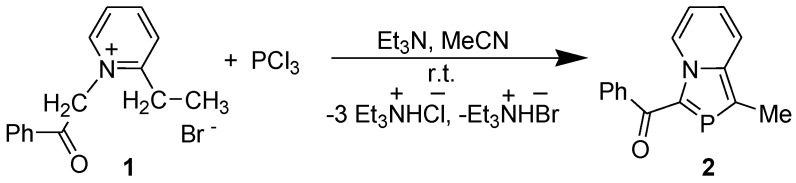
Synthesis of 2-phosphaindolizine derivative.

The reaction of 2-ethyl-1-phenacylpyridinium bromide (**1**) with PCl_3_ in the presence of Et_3_N at room temperature (r.t.) afforded 3-benzoyl-1-methyl-1,3-azaphospholo[5,1-*a*]pyridine (**2**) in very good yield (Scheme 1). The general skeleton was named as 2-phosphaindolizine perceiving it to result from a CH/P exchange at the 2-position of indolizine nucleus [15].

**Scheme 2:**
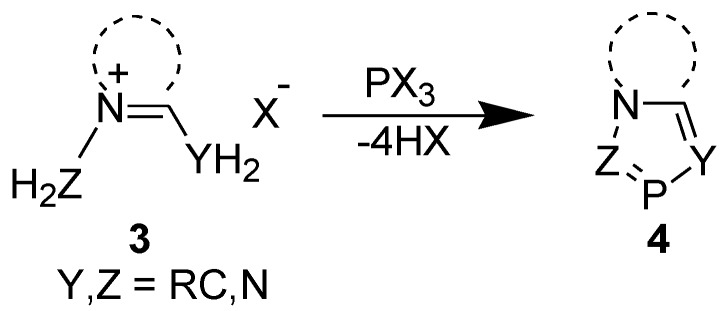
Synthesis of annelated 1,3-azaphospholes.

Later, this synthetic strategy made accessible not only variously substituted 2-phosphaindolizines [15,16,17], but also 1-aza-2-phosphaindolizines [18], 3-aza-2-phosphaindolizines [19], and 1,3-diaza-2-phosphaindolizines [20]. Besides, the method could be extended to the synthesis of 1,3-azaphospholes annelated to pyrazine [15], thiazole [21], benzothiazole [21], and oxazole [22] skeletons also (Scheme 2). These reactions have been compiled in a mini-review [23].

### 2.2. Synthesis Involving Tandem Pyridinium Ylide generation, Disproportionation, and 1,5-Electrocyclization

**Scheme 3:**
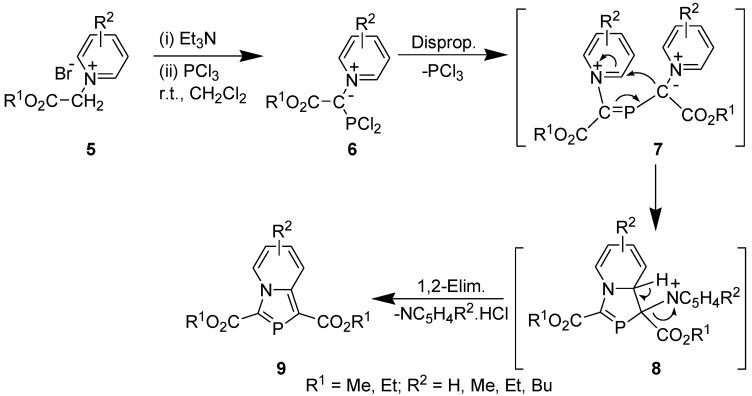
Synthesis of 1,3-bis(alkoxycarbonyl)-1,3-azaphospholo[5,1-*a*]pyridines.

We obtained 1,3-bis(alkoxycarbonyl)-1,3-azaphospholo[5,1-*a*]pyridines (**9**) serendipitously on reacting (alkoxycarbonyl)methylpyridinium bromide with Et_3_N and PCl_3_. The reaction involved tandem pyridinium alkoxycarbonyl-dichlorophosphinomethylide (**6**) generation, disproportionation, 1,5-electrocyclization, and 1,2-elimination to furnish the final product (Scheme 3) [24,25].

Subsequently, isoquinoline [26], and phenanthridine [27] analogues of **9** could also be prepared.

## 3. Reactions Response

### 3.1. Diels-Alder Reaction

**Figure 1:**
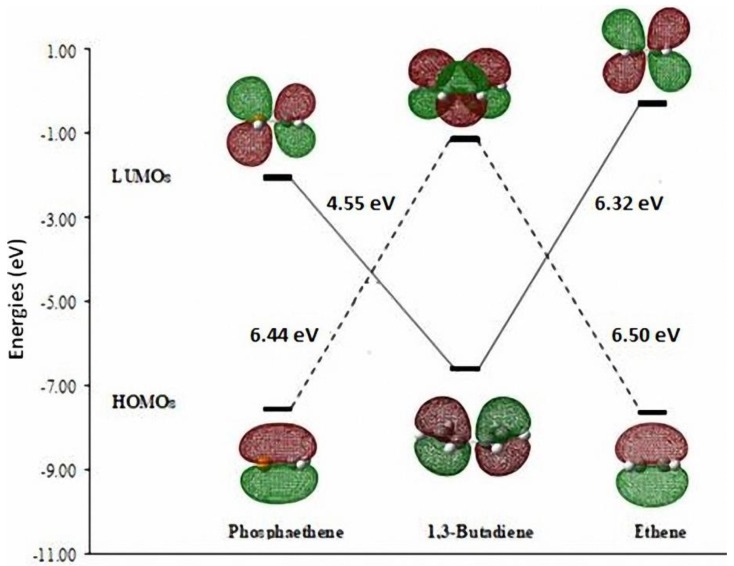
The HOMO-LUMO energy differences between FMOs of phosphaethene, 1,3-butadiene, and ethene calculated at the B3LYP/6-31+G** level.

There have been earlier reports about the Diels-Alder (DA) reaction across the >C=P- functionality as dienophile [28,29]. We investigated theoretically the model DA reactions of ethene and phosphaethene with 1,3-butadiene at the DFT(B3LYP/6-31+G**) level [30]. The Frontier Molecular Orbitals (FMO) of the reactants are shown in Figure 1.

**Scheme 4:**
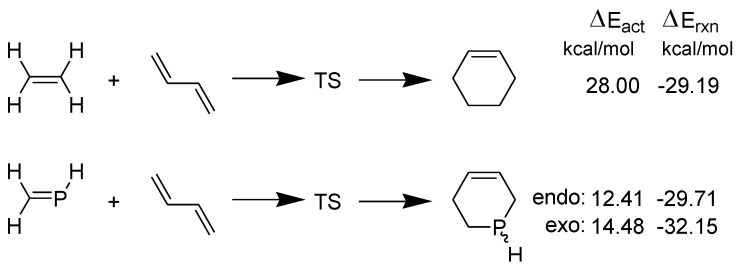
Model DA reactions of ethene and phosphaethene with 1,3-butadiene computed at B3LYP/6-31+G** in the gas phase.

It may be noted that the HOMO_diene_–LUMO_phosphaethene_ gap (4.55 eV) is much lower than the gap HOMO_diene_–LUMO_ethene_ (6.32 eV). Furthermore, the activation energy barriers (ΔEact.) for the two DA reactions are shown in Scheme 4 [30].

**Scheme 5:**
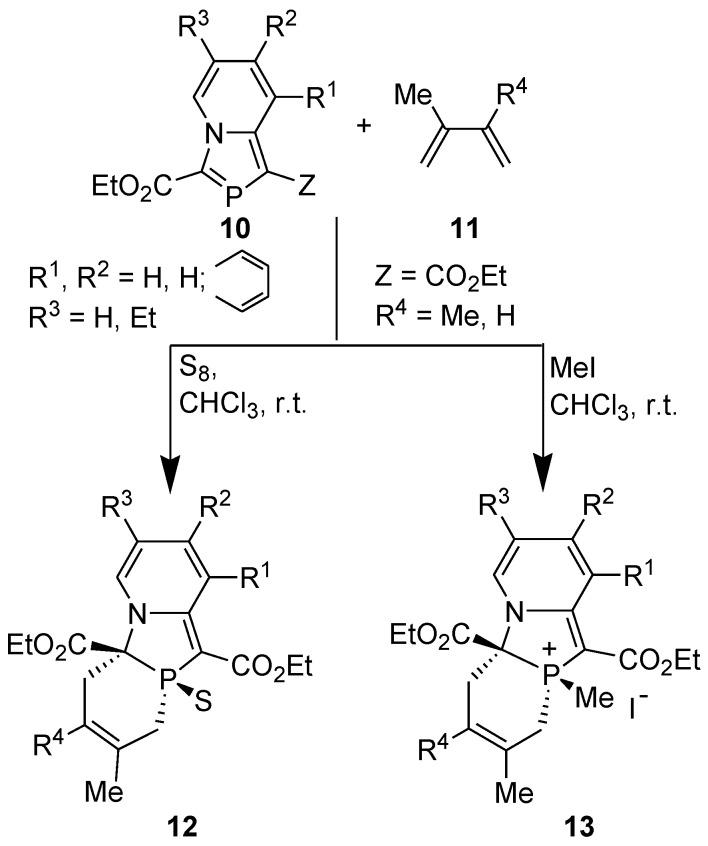
DA reactions of 1,3-bis-(ethoxycarbonyl)-1,3-azaphospholo[5,1-*a*]pyridines with 2,3-dimethyl-1,3-butadiene (DMB ) and isoprene.

Encouraged by these theoretical results, we attempted DA reactions of 1,3-azaphospholo[5,1-*a*]pyridines, i.e., 2-phosphaindolizines prepared by two methods. However, to our great surprise, the compounds 3-ethoxycarbonyl-1-methyl-1,3-azaphospholo[5,1-*a*]pyridine **10a** (**10**, Z=Me, R^1^=R^2^=R^3^=H) obtained through [4+1] cyclocondensation and 1,3-bis(ethoxylcarbonyl)-1,3-azaphospholo[5,1-*a*]pyridine **10b** (**10**, Z=CO_2_Et, R^1^=R^2^=R^3^=H) showed remarkable difference in their reactivity with 2,3-dimethylbuta-1,3-diene (DMB). It was found that 3-(ethoxycarbonyl)-1-methyl-1,3-azaphospholo[5,1-*a*]pyridine (**10a**) did not undergo DA reaction with DMB, even upon boiling in toluene in the presence of sulfur (used to oxidize phosphorus atom of the initially formed cycloadduct to push the reaction in the forward direction, see later) [31]. But, 1,3-bis(ethoxylcarbonyl)-1,3-azaphospholo[5,1-*a*]pyridine (**10b**) underwent DA reaction with DMB at room temperature (r.t.). However, the reaction at room temperature (ca 25 °C) was very slow and could be completed (δ ^31^P NMR = 14.1 ppm) after heating at reflux temperature in chloroform for four days. On carrying out the reaction in the presence of sulfur or methyl iodide, it was complete at r.t. in 4 days [31]. Thus, the DA reactions of 1,3-bis(ethoxycarbonyl)-1,3-azaphospholo[5,1-*a*]pyridines and also of an isoquinoline analogue with DMB and isoprene could be accomplished successfully (Scheme 5) [31,32].

**Scheme 6:**
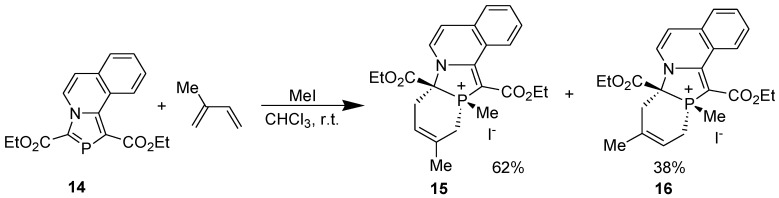
Regioselectivity in the DA reaction of 1,3-bis(ethoxycarbonyl)-1,3-azaphospholo[5,1-*a*]isoquinoline.

All the reactions occurred with complete stereo- and regioselectivity, except the reaction of 1,3-bis(ethoxycarbonyl)-1,3-azaphospholo[5,1-*a*]isoquinoline (**14**) with isoprene in the presence of methyl iodide which afforded two regioisomers, the major (62%) being **15** (Scheme 6) [32].

**Scheme 7:**
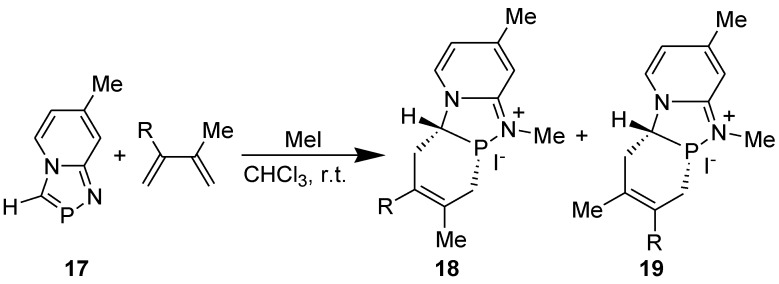
DA reaction of 7-methyl-1,4,2-diazaphospholo[4,5-*a*]pyridine in the presence of methyl iodide.

The structure of the cycloadduct **12** (R^1^R^2^= CH=CH-CH=CH-; R^3^=R^4^=H) was confirmed by single crystal X-ray diffraction studies [32]. That the [2+4]cycloaddition of annelated 1,3-azaphosphole with DMB precedes the oxidation of the phosphorus atom of the initially formed cycloadduct by sulfur or methyl iodide could be proved unambiguously by carrying out the DA reaction of 1,4,2-diazaphospholo[4-*a*]pyridine derivative **17** in the presence of methyl iodide, when N-methylated product **18** (R=Me) was formed (Scheme 7) [33]. It has been reported that [1,4,2]diazaphospholo[4-*a*]pyridines did not react with methyl iodide [34].

**Figure 2:**
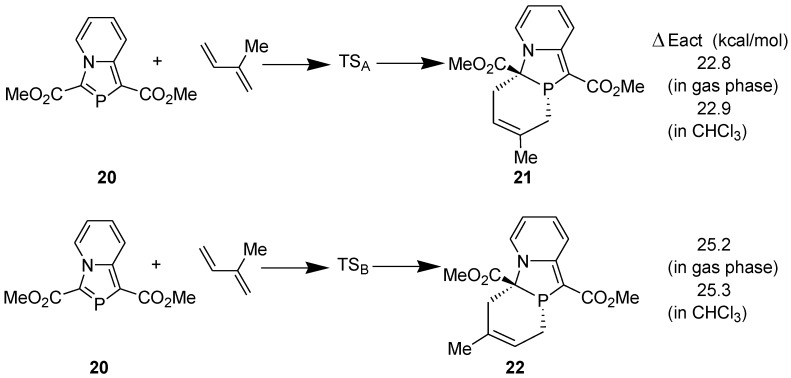
Model DA reactions computed at B3LYP/6-311++G**//B3LYP/6-31G**.

The regioselectivity observed in the reaction of 1,3-bis(ethoxycarbonyl)- 1,3-azaphospholo[5,1-*a*]pyridine with isoprene was investigated theoretically at the DFT (B3LYP/6-311++G**//B3LYP/6-31G**) level. Following model reactions were computed (Figure 2). For studying the solvent effect, single point energy was computed using gas phase-optimized geometry at the B3LYP/6-311++G** level with polarizable continuum model (PCM). 

The regioselectivity was calculated by using the formula, k_1_/k_2_ = e^−∆E/RT^ [35]. A difference of 2.4 kcal/mol in the activation energies corresponds to only 2% regioselectivity. It is obvious that the observed regioselectivity of 100% cannot be accounted for at this theoretical level.

**Scheme 8:**
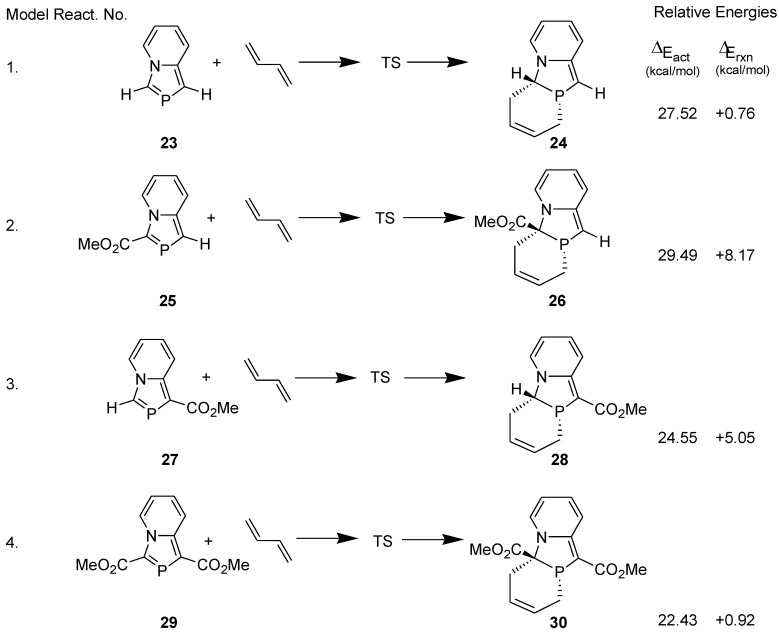
Model DA reactions computed at the B3LYP/6-31G** level.

We investigated the reason for the difference in the dienophilic reactivities of 3-ethoxycarbonyl-1-methyl-1,3-azaphospholo[5,1-*a*]pyridine (**10a**) and 1,3-bis(ethoxycarbonyl)-1,3-azaphospholo[5,1-*a*]pyridine (**10b**), theoretically. For this purpose, following four model reactions were computed at the DFT (B3LYP/6-31G**) level (Scheme 8) [36].

**Figure 3:**
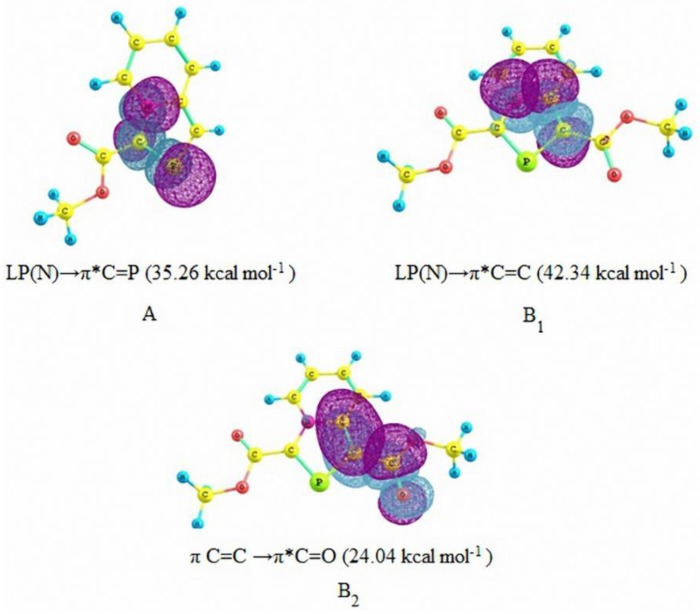
Natural Bond Orbital (NBO) interactions in 3-methoxycarbonyl-1,3-azaphospholo[5,1-*a*]pyridine (**A**) and 1,3-bis(methoxycarbonyl)- 1,3-azaphospholo[5,1-*a*]pyridine (**B_1_** and **B_2_**) with second order perturbation energies (in parenthesis).

As expected, the activation energy barrier for the DA reaction with 1,3-bis(methoxycarbonyl)-1,3-azaphospholo[5,1-*a*]pyridine (**29**) is lowest. However, it is interesting to find that the activation barrier for the DA reaction with 3-methoxycarbonyl-1,3-azaphospholo[5,1-*a*]pyridine (**25**) is highest (29.49 kcal/mol), higher than even for the DA reaction with the unsubstituted 1,3-azaphospholo[5,1-*a*]pyridine (**23**). It is rather an unexpected effect of an electron-withdrawing group (EWG). To look into the real cause of this unusual effect, we carried out the natural bond orbital (NBO) analysis calculations, which revealed the existence of an interesting phenomenon: due to conjugation of the lone pair of the bridgehead nitrogen in **23**, negative charge density is effectively transferred to the five-membered azaphosphole ring, which is further enhanced by the presence of an EWG at the 3-position. It is shown in Figure 3A. It makes the >C=P- functionality electron-rich, which fails to react with electron-rich diene, DMB. However, a CO_2_Me group at the 1-position acts as a trap for the negative charge, the latter being delocalized over to the carbonyl group. It is shown in Figure 3B. Thus in this case, the >C=P- functionality remains unaffected and is able to undergo the DA reaction with DMB.

### 3.2. Lewis Acid Catalyzed Diels-Alder Reaction

**Scheme 9:**
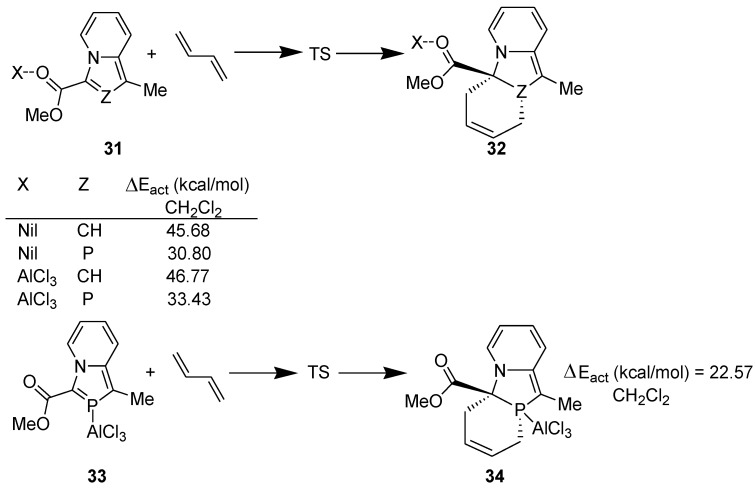
Model DA reactions computed at the B3LYP/6-31+G** level.

Then, we explored the possibility of using a Lewis acid as catalyst to accomplish DA reaction with 3-(alkoxycarbonyl)-1-methyl-1,3-azaphospholo[5,1-*a*]pyridines and computed the following model reactions at the DFT (B3LYP/6-31+G**) level (Scheme 9) [37]. For studying the solvent effect, single point energy was computed using gas phase-optimized geometry with PCM solvation model.

There was another surprise in store for us. When the catalyst, AlCl_3_, is coordinated to the carbonyl group of indolizine or 1,3-azaphospholo[5,1-*a*]pyridine, the activation energy barrier is increased further as compared with that in the absence of the catalyst. The NBO studies reveal that coordination of AlCl_3_ with the carbonyl group enhances transfer of the negative charge in these cases, making the >C=C< and >C=P- functionality in indolizine and 1,3-azaphospholo[5,1-*a*]pyridine, respectively, electron-rich. This appears to be the reason why no DA reaction across the >C=C< moiety of indolizine has been reported even in the presence of a catalyst.

**Scheme 10:**
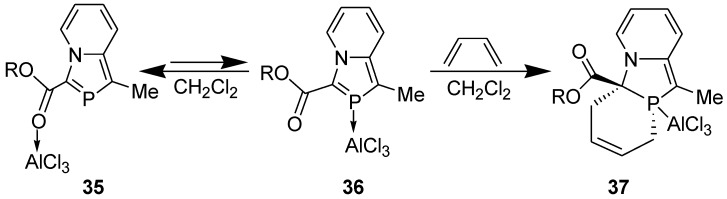
Equilibrium between C=O → and P → coordinated species, and DA reaction with the latter.

In 2-phosphaindolizine, however, there is another site for the coordination of AlCl_3_: the P atom. It may be noted that on coordination of the catalyst to the P atom, the activation energy barrier is lowered remarkably, to 22.57 kcal/mol (in CH_2_Cl_2_) only. These results indicated that in contrast to indolizine, DA reactions across the >C=P- moiety of 2-phosphaindolizine having EWG at 3-position only should be possible in the presence of a Lewis acid catalyst. However, in this context a question arises: if coordination of AlCl_3_ to the carbonyl group is thermodynamically more favorable than its coordination to the P atom by 3.43 kcal/mol, why should it coordinate to the P atom at all? In our opinion, in solution, an equilibrium may exist between the C=O- → AlCl_3_ and P → AlCl_3_ coordinated species, making a small concentration of the latter available that undergoes DA reaction with the diene to produce irreversibly the cycloadduct, thereby shifting the equilibrium to the right (Scheme 10).

**Scheme 11:**
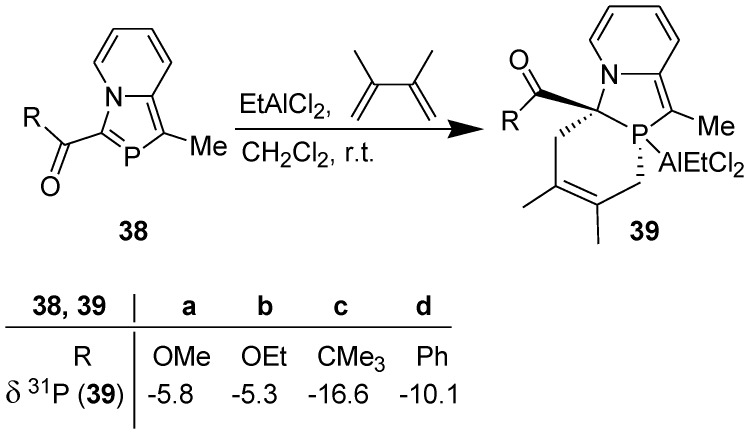
DA reaction of 3-(alkoxycarbonyl/acyl)-1-methyl-2-phosphaindolizines in the presence of EtAlCl_2_.

Guided by the above results, we accomplished successfully the DA reaction of 3-(alkoxycarbonyl/acyl)-1-methyl-2-phosphaindolizines (**38**) with DMB in the presence of ethylaluminium dichloride as a promoter (i.e., using its 1 equiv.) in dichloromethane at r.t. under nitrogen atmosphere (Scheme 11) [38]. The occurrence of a clean reaction was revealed by ^31^P NMR signal at δ = −16.6 to −5.3 ppm, which is in accordance with the ^31^P NMR chemical shifts reported for the [2+4] cycloadducts obtained from the DA reaction of P-W(Co)_5_ complexes of λ^3^-phosphinines [39].

### 3.3. Asymmetric Diels-Alder Reaction with 2-Phosphaindolizines

The successful results described above motivated us to investigate asymmetric DA reaction across the >C=P- moiety of 2-phosphaindolizines by using a chiral organoaluminium catalyst, namely, o-menthoxyaluminium dichloride [40].

**Scheme 12:**
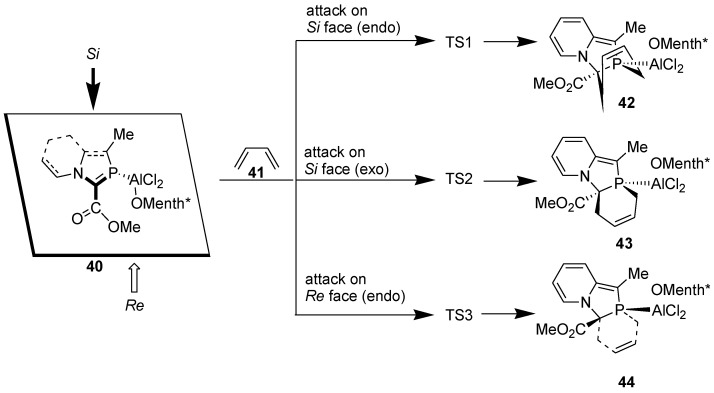
Attack of 1,3-butadiene on *Si* and *Re* faces of the >C=P- functionality of 2-phosphaindolizine complex computed at the B3LYP/6-31+G* level.

We computed the following model reactions at the B3LYP/6-31+G* level (Scheme 12).

Computational calculations reveal that attack of the diene occurs preferentially from the *Si* face. Furthermore, during attack on the *Si* face, activation energy barrier for the *exo* approach is smaller than for the *endo* approach by ca. 0.3 kcal/mol. Presence of the bulky o-menthoxy group possibly makes the *endo* approach comparatively less accessible. All the reactions are moderately exothermic, but due to decrease in entropy, they are endergonic, Gibbs free energies values being +11.65 to +13.08 kcal/mol.

**Scheme 13:**
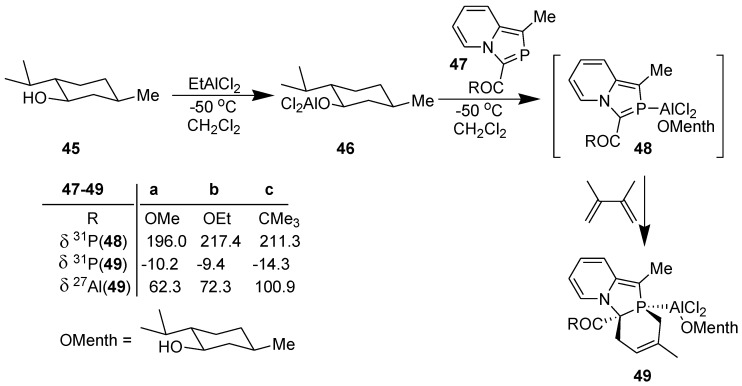
Diels-Alder reaction of 2-phosphaindolizine-n^1^-P-aluminium(*o*-menthoxy) dichloride with DMB.

In accordance with the theoretical results, DA reaction of 2-phosphaindolizines (**47**) could be accomplished with complete diastereoselectivity, as indicated by ^31^P NMR, with a single product being formed in each case (Scheme 13) [40].

### 3.4. Electrophilic Substitution

**Scheme 14:**
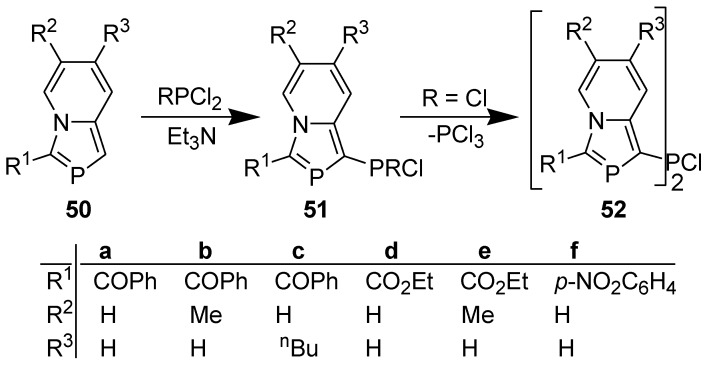
Reaction of 1-unsubstituted 2-phosphaindolizines with chlorophosphines.

In contrast to indolizines [41], 1-unsubstituted 2-phosphaindolizines failed to react with MeCOCl, PhCOCl, or Me_3_SiCl, even on prolonged heating in the presence of Et_3_N [42]. However, like indolizines [43], 1-unsubstituted 2-phosphaindolizines reacted with PCl_3_ or PhPCl_2_ (but not with Ph_2_PCl) in the presence of Et_3_N to afford the phosphynated products (Scheme 14) [44]. It is noteworthy that on reacting with PCl_3_, the initially formed dichlorophosphino product (**51**, R=Cl) undergoes dismutation accompanied by the loss of PCl_3_ to afford a PCl bridged bis(2-phosphaindolizine) (**52**) as the final product.

**Figure 4:**
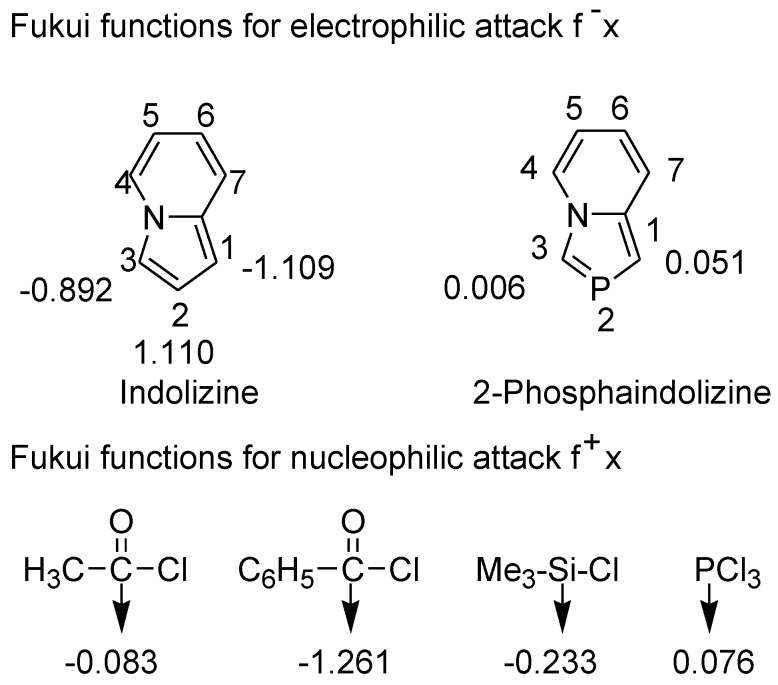
Fukui functions calculated at the B3LYP/6-31+G* level.

The concept of hard-soft acid-base (HSAB) theory was extended to explain the global and local reactivity of organic molecules towards nucleophilic and electrophilic reagents [45]. The chemical reactivity at a particular molecular site could be rationalized using a quantitative descriptor, the Fukui function (f(r)). We recently used Fukui function to rationalize 1,2- versus 1,4-addition of amines to maleic anhydride [46]. The difference in the behavior of indolizine and 2-phosphaindolizine towards electrophilic reagents can be rationalized on the basis of the Fukui functions calculated at the B3LYP/6-31+G* level (Figure 4) [47].

Fukui functions reveal that C1 and C3 in indolizine are much harder than C1 and C3 in 2-phosphaindolizine. Thus, hard electrophilic reagents such as acyl chlorides and chlorotrimethylsilane attack at C1 and C3 positions of indolizine, but they fail to react with 2-phosphaindolizine. On the other hand, phosphorus trichloride is comparatively softer and hence reacts with 2-phosphaindolizine at C1 (C3 being substituted in our compounds). Being at the borderline, PCl_3_ reacts with indolizine also.

## 4. Conclusions

The computational calculations could be used as a useful tool to understand and solve problems encountered during reactions of annelated 1,3-azaphospholers. Furthermore, this strategy worked well while planning the reactions such as Lewis acid catalyzed and asymmetric DA reactions across the >C=P- functionality of 2-phosphaindolizines.
